# Web-Based Assessment of Cognition and Dementia Risk Factors in Over 3000 Norwegian Adults Aged 50 Years and Older: Cross-Sectional PROTECT Norge Study

**DOI:** 10.2196/69867

**Published:** 2025-08-25

**Authors:** Ingelin Testad, Jon Arild Aakre, Martha Therese Gjestsen, Clive Ballard, Anne Corbett, Dag Aarsland, Ellie Pickering, Anastasia Ushakova

**Affiliations:** 1 Centre for Age-Related Medicine - SESAM, Stavanger University Hospital, Stavanger, Norway; 2 College of Medicine and Health, Faculty of Health and Life Sciences, University of Exeter, Exeter, EX1 2LU, United Kingdom; 3 Department of Clinical Medicine, University of Bergen, Haukeland Universitetssykehus, Laboratoriebygget, Bergen, 5009, Norway, 47 48281559; 4 Centre for Healthy Brain Ageing, Department of Psychological Medicine, Institute of Psychiatry, Psychology and Neuroscience, King’s College London, London, United Kingdom

**Keywords:** PROTECT Norge, cognitive function, dementia risk factors, online cohort study, brain health, epidemiology

## Abstract

**Background:**

With the growing number of older adults in the Norwegian population and the associated rapid rise in dementia and cognitive impairment, novel and more efficient methodologies are needed to facilitate research, improve diagnostic triage, and deliver effective brain health interventions in the community. Platform for Research Online to Investigate Genetics and Cognition in Ageing Norge (PROTECT Norge) is a web-based, remote research platform on the aging brain, culturally adapted from the UK PROTECT study, incorporating a Norwegian cohort of adults aged 50 years and older, where participants complete study activities via a dedicated website. Data were collected through study activities, which included self-reported questionnaires and a computerized neuropsychological test battery.

**Objective:**

The study aimed to assess associations between dementia risk factors, including obesity, hypertension, smoking, and hearing loss, and cognition using baseline data from the PROTECT Norge study.

**Methods:**

Data from the PROTECT Norge study were used to assess associations between dementia risk factors and cognition. These associations were assessed using independent-sample *t* tests for each of the cognitive tests, which included paired associate learning, self-ordered search, digit span, and verbal reasoning tasks.

**Results:**

During the first 2 years of data collection, records from 3214 participants were obtained. Associations between established risk factors and cognitive performance were found, with significant detriments to cognitive performance on the computerized neuropsychological test battery. In the PROTECT Norge cohort, consisting of 74.5% females, the mean age was 64.1 (SD 7.7), and 94% of participants provided consent for contact regarding future research.

**Conclusions:**

These data show the associations between dementia risk factors and cognition and attest to the excellent feasibility of the PROTECT Norge cohort, with over 3000 participants included over a period of 2 years and accessibility for people with early cognitive impairment. This, combined with the cohort’s willingness to participate in future studies (94%), positions PROTECT Norge as a tremendous opportunity for cost-efficient, large-scale brain health research and potentially for clinical digital cognitive health programs.

## Introduction

With an aging population, developing new approaches to improve brain health and prevent cognitive decline is essential to empower the community and mitigate against a potential “dementia epidemic.” For example, there are already more than 50 million people worldwide living with dementia, and this is projected to rise rapidly over the next 30 years [1]. Aging is the single biggest risk factor for dementia [2]. In addition, genetic predispositions contribute significantly to dementia risk, both of which are factors with limited potential for mitigation. However, within the domains of lifestyle, mental health, and physical health, there exists a spectrum of potential modifiable risk factors for dementia [3], which are thought to account for 40% of the total dementia risk. With no cure for dementia, addressing these potentially modifiable risk factors through proactive management and preventative interventions is proposed as a key strategy going forward. This approach aims to maintain and improve cognitive health in aging and slow the onset or progression of dementia, to address the expected increase of dementia cases in the population [3,4]. As populations worldwide grow older, the urgency of dementia prevention becomes increasingly evident. In Norway, this trend is reflected in a projected 30.1% increase in those aged 50 years and older from 2024 to 2050 [5], with rural municipalities seeing the greatest impact, where individuals aged 70 years and older could make up a third of the population [6]. Digital technologies show promise in supporting health promotion and disease prevention, with adults aged 50 years and older increasingly integrating them into their daily lives for this purpose [7].

In the United Kingdom, a web-based aging cohort was set up in 2014 to address these challenges. The Platform for Research Online to investigate Cognition and Genetics in Ageing- United Kingdom (PROTECT-UK [8-10]) study aimed to collate large-scale health and cognitive data from older adults to provide novel insights into cognitive trajectory in aging and the risk factors associated with decline. PROTECT-UK (University of Exeter) was built on emerging strengths in digital technologies and the rapid increase in engagement with the internet, computers, and smartphone devices by people in mid and later life. PROTECT-UK demonstrated the enormous potential for this approach to longitudinal research, enabling rapid recruitment of 20,000 participants within the first year, with strong year-on-year retention, for a fraction of the cost of traditional in-person cohort methodologies. Norway was the first country outside the United Kingdom to adopt the PROTECT model, contributing to its international expansion, with Canada later implementing a similar approach [11]. By providing large-scale, standardized cognitive data across multiple countries, PROTECT enables cross-national comparisons and enhances global understanding of dementia risk factors.

Platform for Research Online to Investigate Genetics and Cognition in Ageing Norge (PROTECT Norge) builds upon the PROTECT-UK infrastructure but is tailored to the Norwegian context. PROTECT Norge was launched in September 2020 and has already collected data on more than 5000 participants [12]. The PROTECT infrastructure has enabled large-scale collection of longitudinal data on a range of lifestyle, physical health, mental health, and well-being, in addition to robust, well-validated computerized neuropsychology data in the Norwegian population, providing an accessible, engaging, and cost-efficient approach to cognitive health research using a web-based, remote methodology, which combines computerized cognitive tests and health questionnaires on a dedicated web-based platform with remote genetic testing. The platform provides the opportunity to engage people at scale with epidemiological studies, genomic and biomarker research, and clinical trials. Upon registration, potential participants are given the option to voluntarily consent to being recontacted for future studies, which will enable us to capitalize on their participation in clinical trials relevant to cognition, health, and well-being. For example, this will be an important vehicle to increase the proportion of people who are offered participation in clinical studies, contributing to reach the Government’s action plan and the national target of including 5% of the patient population in Norwegian hospitals by 2025.

The need for such digital initiatives like PROTECT Norge is underscored by the introduction of antiamyloid antibodies for dementia, whose effectiveness depends on early diagnosis. However, this remains a major challenge, as only 1 in 3 dementia cases in the Norwegian community are diagnosed, and diagnosis often occurs in late stages of the condition when irreversible cognitive decline has already led to pronounced functional impairments in daily life and an increased need for care [13].

PROTECT Norge is the only study of this scale incorporating remote data collection, including longitudinal cognitive testing in Norway. The platform is uniquely situated to optimize approaches for the early identification of people with progressive cognitive decline and provide a potential opportunity to evolve as a primary care and community health delivery pathway and resource. While PROTECT Norge is tailored to the Norwegian context, its findings contribute to the wider understanding of cognitive aging and dementia risk factors, supporting ongoing research efforts in dementia prevention and early detection.

This study presents information regarding the baseline characteristics, neuropsychological performance, and risk factors for dementia in the PROTECT Norge cohort with the objective of assessing associations between previously identified dementia risk factors and cognition.

## Methods

### Design

This is a cross-sectional analysis of data collected through PROTECT Norge, a web-based observational cohort study focusing on the aging brain of individuals aged 50 years and older living in the community in Norway. All data are collected remotely via the PROTECT Norge study website. The reporting of the study follows the Strengthening the Reporting of Observational Studies in Epidemiology (STROBE) [14] guidelines and the Checklist for Reporting Results of Internet E-Surveys (CHERRIES) [15] (the CHERRIES checklist is provided in Checklist 1).

### Cross-Cultural Adaptation of PROTECT Norge

All questionnaires without an existing Norwegian translation, along with cognitive tests, underwent a thorough translation process [16]. Two native Norwegian speakers independently translated each measure, 1 familiar with the content and 1 unfamiliar. They then collaborated to create a consensus version. This was back-translated by 2 bilingual translators, preferably native English speakers. Where this was not feasible, Norwegians fluent in English with relevant academic or professional experience were used. An expert committee, including translators from all stages and PROTECT Norge working group members, reviewed all versions and finalized the Norwegian adaptations. For medical and mental health-related questionnaires, additional domain experts and professional translators contributed to quality assurance.

The PROTECT Norge platform underwent multiple rounds of user acceptance testing (UAT). Initial alpha and beta testing of an unscripted manner was conducted by PROTECT-UK and PROTECT Norge personnel. A scripted UAT was then carried out with 30 participants, including end users, user representatives, researchers, and PROTECT Norge personnel. This allowed for simultaneous platform testing and translation validation, ensuring clarity and usability [17].

### Participants

Participants fulfill the inclusion criteria for PROTECT Norge, with all participants aged 50 years or older, residing in Norway, without a diagnosis of dementia, and with a good understanding of the Norwegian language. Participants must also have access to a computer or tablet with an internet connection. The vast majority of people over the age of 50 years in Norway are eligible, thus providing an opportunity for the whole community within this age group to participate in research. Participation is entirely remote and can be completed at home.

### Recruitment

Participants were recruited to PROTECT Norge through publicity in national and regional broadcasting channels, newspaper coverage, placement of written materials in health care facilities, and oral presentations. However, social media, particularly Facebook, served as a primary recruitment channel through both nontargeted posts and targeted advertisements, the latter applying demographic filters based on the inclusion criteria (age 50 years and older and residing in Norway). The participants register, confirm eligibility, and provide consent through an ethically approved, validated web-based procedure, which includes voluntary consents for contact for future research and consent for providing saliva for genetic sampling. Following registration, participants provide demographic information and then receive email reminders for assessments and study activities. Participants complete an annual assessment of validated questionnaires and assessments on the PROTECT Norge platform.

### Cognitive Assessments

Participants complete an annual cognitive assessment using a validated computerized cognitive assessment system. This analysis presents data from 4 widely used and well-validated cognitive tests that were in use on the platform from 2020 to 2022, including tests of spatial working memory (Paired Associate Learning [18], Self-Ordered Search [19]), numerical working memory (Digit Span [20]), and executive function (Verbal Reasoning [21]). The Trail-making B test was also included to enable benchmarking against well-established cognitive norms [22]. Mild cognitive impairment (MCI) was identified based on established thresholds [23].

Although not presented in this paper, it is important to note that in 2022, the cognitive assessment protocol was updated to the FLAME (Factors of Longitudinal Attention, Memory, and Executive Function; University of Exeter) cognitive test battery which includes the 4 original tests in addition to 3 tests of reaction time and attention, and 1 of attention and episodic memory. The FLAME battery is well validated [23], showing good discrimination between different levels of cognitive impairment, good sensitivity to change, and good concurrent validity with functional change. Importantly, the battery is validated for self-test in their own home [10].

### Health Questionnaire Assessments

Participants complete annual questionnaire assessments to capture key risk and cognitive association factors. Participants provide information regarding current and past medical conditions through a tick-box selection of key conditions, including hypertension and hearing loss, free-text capture of prescribed medications, and height and weight to enable BMI calculation. Participants also complete a comprehensive questionnaire set to capture lifestyle and modifiable risk factors, including questions on smoking status (current-, ever-, and nonsmoker). Where participants have voluntarily nominated an informant, the informant is invited to complete adapted versions of specific questionnaires pertaining to the participant. Although not evaluated as part of the current paper, the other assessments undertaken are summarized in Textbox 1 for information.

Textbox 1. **Other assessments completed in PROTECT Norge (Platform for Research Online to Investigate Genetics and Cognition in Ageing Norge).**

The text box lists other study assessments undertaken by participants in PROTECT Norge:

- Health Questionnaire: captures Medical conditions and prescribed medications, sensory impairments, pain, and history of traumatic brain injury
- Family history of neurodegenerative disease questionnaire
- The Informant Questionnaire on Cognitive Decline in the Elderly (IQCODE): self and informant questionnaire
- Mild Behavior Impairment (MBI) scale: measures neuropsychiatric symptoms; available as self and informant questionnaire
- Instrumental Activities of Daily Living questionnaire
- Lifestyle questionnaire: includes items capturing use of technology, cognitive training, alcohol use, caffeine intake, dietary supplement use, use of cognitively stimulating activities, and the number of languages spoken, physical activity, including CHAMPS (Community Healthy Activities Model Program for Seniors), physical activity questionnaire for older adults. This contains adaptive questioning, whereby certain items are conditionally displayed based on previous responses
- Composite mental health questionnaire: Patient Health Questionnaire (PHQ-9) and General Anxiety Disorder (GAD-7) scales as assessments of depression and anxiety, history of mental health disorder, items from the World Health Organization (WHO) composite international diagnostic interview psychosis and manic modules, addictions, including Alcohol Use Disorder Identification Test (AUDIT), questions relating to self-harm, suicidality, well-being, and experiences in adult life and childhood. Items exploring neurodivergent symptoms from childhood. This questionnaire is voluntary in its entirety, and participants may choose “prefer not to answer” for any item. It also uses adaptive questioning
- Sleep: St. Mary’s Sleep Questionnaire, Insomnia Severity Index, Bergen Insomnia Scale, and Epworth Sleepiness Scale
- Awareness of age-related change-10 SF: assesses perceived gains and losses with aging
- University of California, Los Angeles Three-Item Loneliness Scale (UCLA-3)
- ASK (Aging, Social Dynamics, and Knowledge) questionnaire: captures information about living situations, social interactions, technology use, travel habits, future housing preferences, and views on future care models

For all questionnaires, respondents can review and modify their responses at any time before final submission.

### Patient and Public Involvement

User involvement is paramount in all health research and aligns with the mission of the Ministry of Health and Care Services to regional health authorities and has been the core value in all activities at the Centre for Age-Related Medicine - SESAM since its establishment in 2010. PROTECT Norge is supported by 2 designated user representatives who have been actively engaged since the start of PROTECT Norge. They are part of the steering group, have been represented in seminars, and have been actively involved in the translation of material from PROTECT-UK.

### Statistical Analysis

Categorical variables were described using count and percentage. Continuous variables were described using mean (SD) if symmetrically distributed and median with IQR if otherwise. For each test, the average value of all scores over the number of times it was completed (from 1 to 3 at each testing time point) was used as a final score.

For each cognitive test score and each potential medical risk factor (Hypertension–present or absent, smoking ever, or nonsmoker), BMI (<30 v>30), hearing loss—present or absent—identified as risk factors from the Lancet commission meta-analysis [3], we performed a 2-tailed independent sample *t* test for each of the cognitive tests (df=3212). Two-tailed *P* values <.05 were considered significant; no adjustments for confounders and no correction for multiple testing were done. All statistical analyses were undertaken using SPSS (version 29; IBM Corp).

### Ethical Considerations

This study uses data from the PROTECT Norge Study [12], launched in September 2020 and approved by the Regional Committee for Medical and Health Research Ethics, West (2019/478), and the study has been approved by the Stavanger University hospital. Informed consent was obtained through a validated web-based procedure, and data are stored in compliance with the General Data Protection Regulation (GDPR) and Stavanger University Hospital’s data security policy [24]. To protect privacy, data were pseudonymized before analysis. No compensation was provided for participants, except for gaining access to brain training games on the PROTECT Norge platform.

## Results

### Cohort Characteristics

During the first 2 years of the data collection period, 3214 participants completed the baseline assessment. The average age of the cohort was 64 years, of whom 74.5% (n=2393) were female and nearly 80% (n=2503) had a higher education degree. The majority (n=2289, 71.7%) of the participants were either widowed, divorced, or single, and 50% reported that they were working either part- or full-time. Of all the participants (n=3214), 58 had MCI. The full cohort characteristics are shown in Table 1.

The majority of participants (74.4%) reported hearing about PROTECT Norge through social media. Additional sources included friends and acquaintances (10.3%), traditional media (TV, radio, and newspapers; 4.4%), other study participants (4.2%), other sources (5.8%), and courses or conferences (0.9%). Consent for contact for future research was provided by 94% of the participants in PROTECT Norge.

**Table 1.** Characteristics of PROTECT Norge^a^ cohort participants.

Characteristics (n=3214)^b^	Values
Age (years), mean (SD), min/max	64.1 (7.7), 50.0/89.2
Female, n (%)	2393 (74.5)
Higher education, n (%)	2503 (77.9)
Marital status, n (%)	
Married, civil partnership, or cohabiting	2289 (71.7)
Widowed, separated, divorced, or single	902 (28.3)
Working (full-time or part-time), n (%)	1608 (50.2)
High blood pressure, n (%)	656 (38.3)
Mild cognitive impairment, n (%)	58 (1.8)
Hearing loss, n (%)	818 (29.6)
Smoking, n (%)	
Current smoker	153 (5.6)
Stopped smoking	1465 (53.2)
BMI (reference: normal), n (%)	
Underweight	24 (0.9)
Overweight	1057 (38.3)
Obese	374 (13.6)
Very obese	26 (0.9)

a PROTECT Norge: Platform for Research Online to Investigate Genetics and Cognition in Ageing Norge.

b This table presents the demographic and health-related characteristics of the participants in the PROTECT Norge cohort study.

### Cognitive Profile of the Cohort

The mean scores for each of the neuropsychological tests undertaken are described and compared between participants with and without MCI, showing that performance was significantly more impaired on the 4 PROTECT tasks in people with MCI compared with those with normal cognition (Table 2).

**Table 2.** Neuropsychological test scores and comparison of cognitive performance between people with MCI^a^ and healthy cognition.

Test^b^	Mean (SD)	MCI vs normal cognition	
		*t* test (*df*)	*P* value
Digit Span Memory Test	6.3 (2.5)	6.7 (3212)	<.001
Paired Associate Learning	3.6 (1.0)	3.3 (3212)	<.001
Verbal Reasoning task	24.5 (10.1)	8.8 (3212)	<.001
Self-Ordered Search task	33.9 (14.4)	6.3 (3212)	<.001

a MCI: mild cognitive impairment.

b This table presents the mean scores (SD) on neuropsychological tests among individuals with mild cognitive impairment (MCI) compared to those with normal cognition. The statistical comparison between the MCI group and the normal cognition group is shown with corresponding *t* values (df=3212) and *P* values.

### Association of Medical Risk Factors With Cognitive Performance

A consistent pattern of mainly significant associations was seen between the Lancet commission risk factors for dementia examined (hypertension, smoking, hearing loss, and BMI>30) [3] and the neuropsychological test battery scores. The full results are shown in Table 3.

**Table 3.** Association between medical risk factors for dementia and cognitive test scores in PROTECT Norge^a^.

Medical risk factors^b^	Digit span		PAL^c^		Verbal reasoning		Self-ordered search	
	*t* test (*df*)	*P* value	*t* test (*df*)	*P* value	*t* test (*df*)	*P* value	*t* test (*df*)	*P* value
Hypertension	2.3 (3212)	.02	2.7 (3212)	.006	2.4 (3212)	.02	1.5 (3212)	.13
Hearing loss	3.6 (3212)	<.001	3.5 (3212)	<.001	2.9 (3212)	.004	1.7 (3212)	.09
Smoking	3.0 (3212)	.003	2.5 (3212)	.007	2.8 (3212)	.005	2.7 (3212)	.007
BMI >30	1.9 (3212)	.06	1.5 (3212)	.07	4.3 (3212)	<.001	3.1 (3212)	.002

a PROTECT Norge: Platform for Research Online to Investigate Genetics and Cognition in Ageing Norge.

b This table displays the association between various medical risk factors for dementia and cognitive test scores among participants in the PROTECT Norge study. Statistical significance is indicated by *t* values, including degrees of freedom and corresponding *P* values (P).

c PAL: paired associate learning.

## Discussion

### Principal Findings

This study reports the baseline characteristics of the first 3214 participants in the PROTECT Norge digital cohort study of people aged 50 years and older. We found a consistent pattern of mainly significant associations between the Lancet Commission risk factors for dementia [3], including hypertension, smoking, hearing loss, and BMI>30, and lower cognitive performance on the neuropsychological test battery scores.

### Comparison to Previous Work

Established risk factors for dementia (BMI, smoking, hypertension, and hearing loss) were all significantly associated with lower cognitive performance on the computerized neuropsychological tests, which is important for several reasons. First, it acts as a validation of the PROTECT Norge cohort, demonstrating the same pattern of risk associations that have been identified in other international studies [3]. Second, the work builds on this by demonstrating that in addition to the longer-term risks for dementia identified in other studies, these risk factors are associated with subtle but measurable cognitive impairments in individuals who are cognitively healthy but with an increased future risk of dementia. This also highlights the opportunities to improve cognitive health through intervention studies in the Norwegian population. Hearing loss is a relatively newly identified risk factor for dementia, and it is therefore of particular note that a strong association was seen between hearing loss and cognitive test performance. Emerging work also suggests that the risk can be mitigated by wearing hearing aids [25,26], with important potential public health implications.

### Strengths and Limitations

First, a strength of the PROTECT Norge study is its fully digitalized study design, which has enabled the inclusion of over 3000 Norwegian individuals over a period of 2 years, during which high-quality data, including computerized neuropsychology, validated questionnaires collecting health, mental health, and lifestyle data were successfully collected. In addition, 94% of the cohort has consented to be contacted for future research. Second, the computerized neuropsychology cognitive test battery is objectively measured, demonstrates good discrimination between different levels of cognitive impairment, and has been validated for self-testing in a home setting [10]. However, other health assessments used in this study rely on self-reported data, which may introduce potential biases such as recall errors and misestimation. Third, it should be acknowledged that the findings reported in this study are based on cross-sectional data, meaning causality cannot be inferred.

Fourth, the cohort is predominantly Caucasian, female, and well-educated, in line with other digital health studies, and the self-selection recruitment strategy and a heavy reliance on Facebook for outreach have likely contributed to this imbalance, as women in this age group are more engaged on this platform [24,27]. Finally, although it is encouraging that participants with MCI successfully completed study assessments, only 58 of 3214 participants had MCI, well below population estimates and the proportion seen in PROTECT UK.

### Future Direction

In 2030, for the first time in Norway, there will be more people over 65 years than 19 years and younger [6] and the number of people with dementia will increase from 101,118 in 2020 to almost 235,000 in 2050 [28], along with a reduction in the ratio of people working to those who are retired. These numbers are even more dramatic in rural districts [6]. Better brain health and improved initiatives to diagnose early cognitive impairment and prevent dementia will be imperative. Lifestyle and cognitive training interventions have been found to improve brain health and prevent cognitive decline [29,30], and the PROTECT infrastructure is well placed to deliver large-scale interventions to the Norwegian population. Digitalized research and health care platforms will be an essential part of improving the brain health of older people in our society.

This study demonstrates the feasibility of the PROTECT model, highlighting the tremendous opportunity for this approach to contribute significantly to large-scale, cost-effective brain health research and service delivery as we move forward. In addition, despite the current demographic limitations seen in PROTECT Norge, this study still provides valuable insights into how these risk factors influence cognition within a large cohort, contributing to the understanding of cognitive health at a population level. However, future efforts are warranted, specifically to improve demographic diversity in the cohort and increase the representation of individuals with early cognitive impairment. Moving this forward could be done by specifically targeting underrepresented demographic groups on social media platforms, whilst simultaneously expanding outreach beyond social media to relevant interest groups or organizations and collaboration with primary care providers.

### Conclusion

In summary, our study using PROTECT Norge data reveals significant associations between established dementia risk factors, including obesity, hypertension, smoking, and hearing loss, and lower cognitive performance, even among individuals who are cognitively healthy but at increased future risk of dementia. These findings support the validity of the PROTECT model and highlight its potential to detect early cognitive changes in at-risk populations. The successful collection of large-scale, remotely administered data further demonstrates the feasibility of this digital approach. PROTECT Norge offers a unique and cost-effective opportunity to scale brain health research and interventions across Norway, particularly by targeting individuals with modifiable risk factors.

However, to improve cohort representativeness, more targeted recruitment strategies are needed to reduce selection bias and enhance demographic diversity. Future efforts should focus on expanding outreach channels and optimizing participant engagement to improve the inclusion of underrepresented demographic groups, including individuals with MCI.

## Supplementary material

10.2196/69867 Checklist 1 Checklist for Reporting Results of Internet E-Surveys.

## Abbreviations

CHERRIES: Checklist for Reporting Results of Internet E-Surveys
FLAME: Factors of Longitudinal Attention, Memory, and Executive Function
GDPR: General Data Protection Regulation
MCI: mild cognitive impairment
PROTECT Norge: Platform for Research Online to Investigate Genetics and Cognition in Ageing Norge
PROTECT-UK: Platform for Research Online to Investigate Cognition and Genetics in Ageing- United Kingdom
STROBE: Strengthening the Reporting of Observational Studies in Epidemiology
UAT: user acceptance testing
